# Exploiting Urazole’s Acidity for Fabrication of Hydrogels and Ion-Exchange Materials

**DOI:** 10.3390/gels7040261

**Published:** 2021-12-13

**Authors:** Saltuk B. Hanay, Ali Fallah, Efsun Senturk, Zeliha Yetim, Ferdows Afghah, Hulya Yilmaz, Mustafa Culha, Bahattin Koc, Ali Zarrabi, Rajender S. Varma

**Affiliations:** 1 Faculty of Engineering and Natural Sciences (FENS), Sabanci University, Istanbul 34956, Turkey; ali.fallah@sabanciuniv.edu (A.F.); efsunsenturk@sabanciuniv.edu (E.S.); ferdows.afghah@sabanciuniv.edu (F.A.); bahattinkoc@sabanciuniv.edu (B.K.); 2 Sabancı University Nanotechnology Research and Application Center—SUNUM, Istanbul 34956, Turkey; hulya.yilmaz@sabanciuniv.edu (H.Y.); mustafa.culha@sabanciuniv.edu (M.C.); 3 Hanay Advanced Chemicals Inc., Hanay Ileri Kimya Arastirma Gelistirme ve Muhendislik AS, Istanbul 34413, Turkey; 4 Integrated Manufacturing Technologies Research and Application Center, Sabanci University, Istanbul 34956, Turkey; 5 Department of Histology and Embryology, Faculty of Medicine, Ataturk University, Erzurum 25240, Turkey; zelihayetim@hotmail.com; 6 Department of Internal Medicine and Ophthalmology, Morsani College of Medicine, The University of South Florida, Tampa, FL 33620, USA; 7 Department of Biomedical Engineering, Faculty of Engineering and Natural Sciences, Istinye University, Istanbul 34396, Turkey; alizarrabi@gmail.com; 8 Regional Centre of Advanced Technologies and Materials, Czech Advanced Technology and Research Institute, Palacký University in Olomouc, 78371 Olomouc, Czech Republic

**Keywords:** hydrogels, urazole, ion-exchange, porous gels, biomaterials

## Abstract

In this study, the acidity of urazole (pKa 5–6) was exploited to fabricate a hydrogel in two simple and scalable steps. Commercially available poly(hexamethylene)diisocyanate was used as a precursor to synthesize an urazole containing gel. The formation of urazole was confirmed by FT-IR and ^1^H-NMR spectroscopy. The hydrogel was characterized by microscopy imaging as well as spectroscopic and thermo-gravimetric analyses. Mechanical analysis and cell viability tests were performed for its initial biocompatibility evaluation. The prepared hydrogel is a highly porous hydrogel with a Young’s modulus of 0.91 MPa, has a swelling ratio of 87%, and is capable of exchanging ions in a medium. Finally, a general strategy was demonstrated to embed urazole groups directly into a crosslinked material.

## 1. Introduction

Hydrogels have become a very important class of soft materials, with global sales reaching 15–20 billion U.S. dollars annually [1]. Since their first demonstrated application in 1960 [2], hydrogels have been used in many different areas such as contact lenses, wound dressings, cosmetics, drug delivery systems, tissue engineering, agriculture, and hygiene products [3]. Hydrogels can be made from synthetic polymers such as polyethylene glycol (PEG), natural polymers such as gelatin and collagen, or hybrids of natural and synthetic polymers [4]. The choice of polymers and crosslinkers is crucial, as these parameters affect the final properties of the hydrogel. Pore size, mechanical properties, swelling ratio, swelling rate, biodegradability, biocompatibility, chemical resistance, optical properties, and stimuli responsiveness are additional important hydrogel properties, which must be tailored according to the application. For example, a high swelling ratio and fast swelling kinetics are essential for superabsorbent applications [5]. On the other hand, biodegradability and biocompatibility are crucial in drug delivery or tissue engineering applications [6,7].

Hydrophilicity, a vital property for hydrogels, is generally provided by hydrophilic polymers such as PEG or polyacrylamides. Although there are numerous reports on hydrogels, novel formulations that can outperform commercial products are still needed. Sophisticated hydrogel formulations having superior properties may not be sufficient for commercialization. In addition, scalability of the hydrogel synthesis, availability of starting materials, and the cost of the fabrication need to be satisfactory for industrial production. In this paper, we describe a facile method to fabricate a novel hydrogel based on urazole starting from inexpensive and readily available precursors. We also demonstrated the potential use of urazole-based hydrogels as an ion-exchange material.

**Figure 1:**
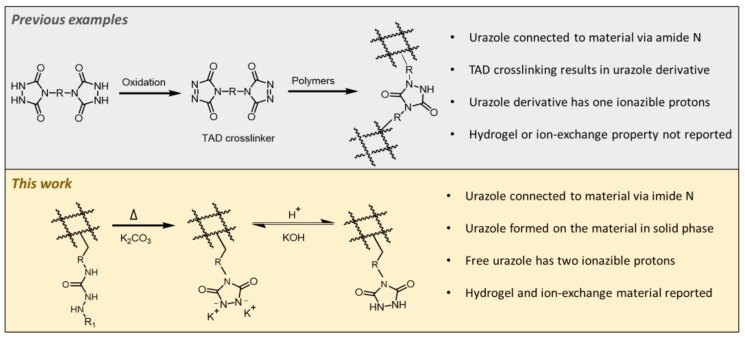
Comparison of urazole containing materials with the present work.

Urazoles are nitrogen-containing heterocycles that have been mostly used as precursors of triazolinediones (TADs) which have recently attracted attention owing to their click and click-like reactions [8,9,10,11,12,13,14]. Urazoles can readily be oxidized to TADs by strong oxidants [15,16]. Alternatively, some of the urazole derivatives can be aerobically oxidized to corresponding TADs by laccase enzyme [17]. Moreover, in situ oxidation of urazoles can be done by using a ruthenium photocatalyst [18]. In general, TAD or in situ oxidized urazole forms adducts where urazoles are bound to a material via urazole’s amide nitrogen (Figure 1). There are only a few reports where urazole is attached to material via its imide nitrogen [19,20]. However, in these reports urazoles were not explored as a main functional group. They were converted into corresponding TADs for further reactions.

Urazole has rarely been considered as a main functional group, as TADs highly successful click reactions have deflected attention from it. However, urazole protons are highly acidic [21,22] and this might be exploited to obtain functional materials possessing anionic character. The acidity of urazole has been studied in detail in proton-transfer reactions [23]. In one study, novel polymers were synthesized via N-alkylation of urazoles by exploiting the acidity of urazole [24]. Urazole derivatives such as 4-oleyl urazole and 1,2-diacetyl-4-oleyl urazole have been patented as additives for functional fluids [25]. In another study, the potential of urazoles as analogues of prostaglandins in bronchodilation was demonstrated [26]. Interestingly, urazole was thought to be a precursor of uracil in the pre-RNA world, as it reacts with ribose to form four different ribosides [27]. Recently, Yang et al. deployed urazole to urazole-gold nanoparticles to determine curcumin using fluorescent spectrometry [28].

Herein, we envisioned a method to obtain an urazole-containing material from inexpensive and commercially available precursors. The process involves embedding urazole’s precursor (semicarbazide) in the polymer backbone rather than attempting to attach urazole directly to a material. The latter strategy is synthetically challenging due to the amide protons of urazole. Semicarbazide was converted to urazole in solid-state under strong basic conditions. The ensuing urazole-containing gel was a hydrogel, with 87% swelling ratio and 0.91 MPa elastic modulus. Interestingly, the polymeric backbone of the material is highly hydrophobic, with the ionic salt character of urazole conferring the hydrophilicity of the material. We also demonstrated that urazole can be exploited as an ion exchanger. It was shown that the hydrogel successfully removed Ca^2+^ and Mg^2+^ ions from tap water while releasing potassium ions. Finally, the high Young’s modulus, low toxicity profile, ion responsiveness, as well as the economic and scalable production method make this material a promising candidate for hydrogel and ion-exchange applications.

## 2. Results and Discussion

**Scheme 1:**
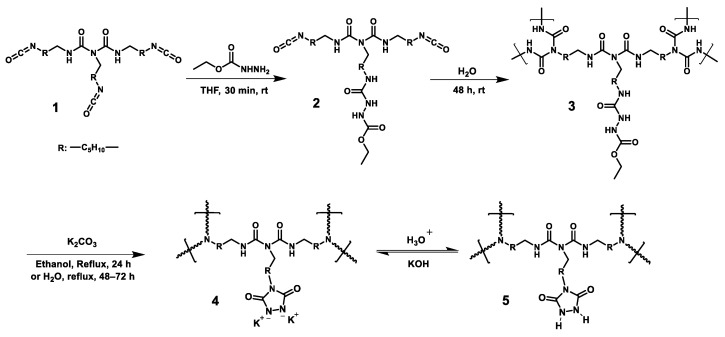
Synthesis of isocyanate-semicarbazide intermediate (**2**), semicarbazide gel (**3**), urazole potassium salt hydrogel (**4**), and urazole gel as free acid (**5**) starting from commercially available poly(hexamethylene diisocyanate) (**1**).

The synthesis of the urazole gel is shown in Scheme 1. In the first step, a multifunctional isocyanate, namely (1) poly(hexamethylene diisocyanate), reacted with equimolar ethyl carbazate to form an isocyanate semicarbazide-containing prepolymer intermediate (2). In the same reaction flask, polymerization of the prepolymer was initiated via the addition of a small amount of water. Polymerization of poly(urea) was also accompanied by crosslinking, a process that is highly sensitive to moisture and heat. High temperatures or open-air conditions resulted in bubbles in the gel (Electronic Supporting Information, ESI Figure S1). However, bubble-free gels were obtained by carrying out the gelation reaction at ambient temperatures and under inert atmosphere (ESI Figure S2). The semicarbazide-containing gel (3) was cyclized into a urazole-potassium gel (4) under strong basic conditions at high temperature. Conversion of semicarbazide to urazole can be conducted using K_2_CO_3_ in ethanol or K_2_CO_3_ in water. Formation of urazole is more efficient and faster when ethanol is used. However, ethanol must be removed from the gel and replaced with water. Therefore, at this step, solvent exchange should be performed carefully and slowly to prevent stress on the gels. Cyclization of urazole with K_2_CO_3_ results in a urazole-potassium gel. This ionic gel can be acidified to obtain a urazole gel in a free acid form (5). Interestingly, aurazole-potassium gel (4) can hold a large amount of water owing to its urazole-potassium salt groups.

**Figure 2:**
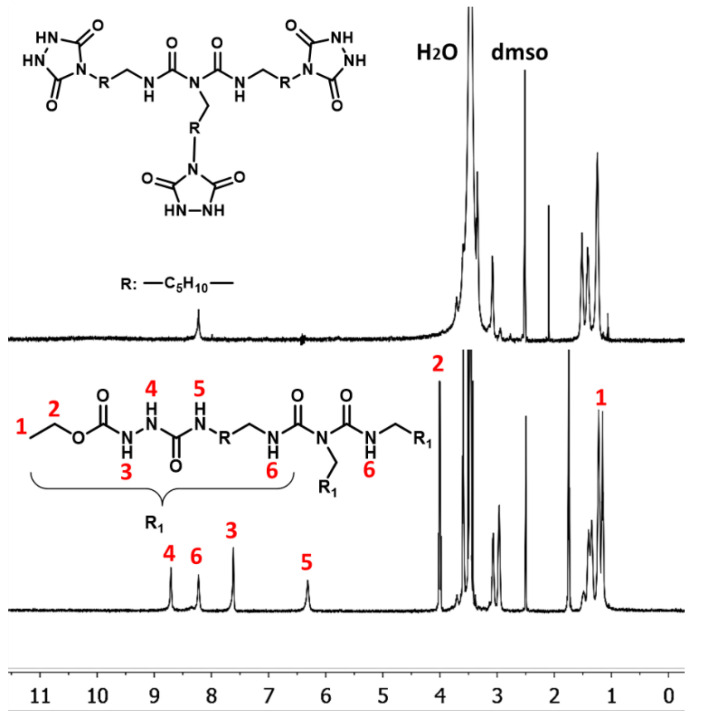
^1^H-NMR spectrum of the model compounds poly(hexamethylene semicarbazide) and poly(hexamethylene urazole). R group represents –C5H10– in each spectrum.

**Figure 3:**
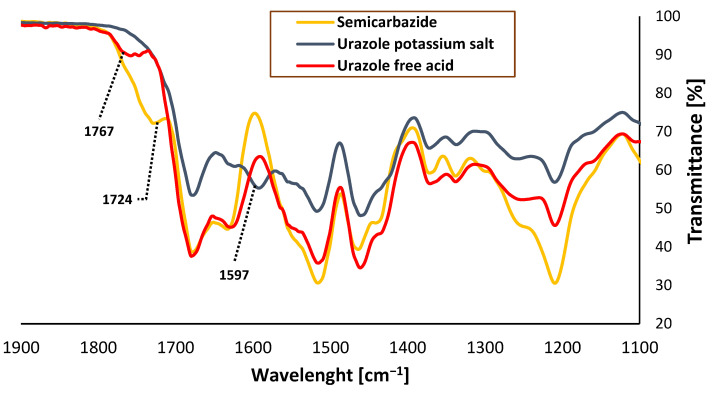
FT-IR spectrum of urazole-potassium gel, urazole free acid gel and semicarbazide gel.

^1^H-nuclear magnetic resonance (NMR) analysis of urazole cyclization was performed using a model compound, as the gels are crosslinked and not soluble. To obtain a soluble model compound, all the isocyanate groups of poly(hexamethylene diisocyanate) were reacted with ethyl carbazate to form poly(hexamethylene semicarbazide) (ESI, Scheme S1). The model compound is soluble in DMSO, and the ^1^H-NMR spectrum analysis showed successful formation of semicarbazide and urazole. (Figure 2) Disappearance of the peaks attributed to semicarbazide (3, 4, and 5) showed that urazole formation had been successful. The synthesized gels were characterized by Fourier Transform Infrared (FT-IR, Figure 3, Figure S6) and Raman spectroscopy (Figure S7). The FT-IR spectra of semicarbazide gel (3) and urazole gel (4) reveal that a new carbonyl mode at 1767 cm^−1^ appeared after urazole formation (Figure 3). Additionally, the carbonyl mode of semicarbazide at 1724 cm^−1^ disappeared upon urazole formation. These FT-IR findings are also in agreement with our previously synthesized urazole compounds where their structures were also confirmed with ^1^H-NMR [29]. Urazole-potassium salt has a unique mode at 1597 cm^−1^ and the carbonyl mode at 1767 cm^−1^ was not present when FT-IR analysis was performed under dry and neat conditions.

Urazole formation was determined indirectly by converting the urazole groups into TAD [30]. Formation of the characteristic red/pink color of TAD can be used to determine the urazole groups qualitatively. The oxidation can be conducted with strong oxidants such as silica bound nitric acid or using 1,3-dibromo-5,5-dimethylhydantoin. There is a possibility that semicarbazide derivatives may form diethyl azodicarboxylate (DEAD)-like structures upon oxidation, which have a similar color (orange to red) and reactivity to TADs. However, semicarbazide gels (3) did not give any color when they were treated with strong oxidizers including HNO_3_ and 1,3-dibromo-5,5-dimethylhydantoin for at least 1 h. This shows that the red color of the gel was formed because of TAD formation instead of a possible DEAD-like byproduct formation.

Quantification of urazole groups can be accomplished indirectly by determining the TAD groups on the gels. Titrating the TAD groups against one of the reactants can be used for quantification. However, the titration method is prone to human error, as reactants take some time to diffuse deep into the gel. NMR analysis is a more reliable method to quantify TADs. A stock solution of a TAD reactive molecule can be used to determine the TAD content of the gel. However, the reactant molecule should be chosen carefully. For example, furan has a low boiling point (30 °C), and some of it could easily escape causing a positive error in TAD quantification. On the other hand, aniline forms 1:2 adducts with TAD that cause a negative error in quantification (ESI, Figures S3 and S4). Additionally, the amine group of aniline can also decompose TAD over time which complicates the quantification. Some compounds such as 1-naphthol also react with more than one TAD group. A reliable analysis could be performed using 2-naphthol, as it forms a 1:1 adduct with TAD (ESI Figure S5). To determine the TAD content of the gels, three different batches of urazole potassium gels were oxidized to TAD. Quantification was done using 2-naphthol as the reactant. ^1^H-NMR results showed that an average of 0.71 mmol of 2- naphthol was reacted indicating the amount of active TAD on the gel (Table 1). For accurate determination, acetonitrile was added to the reactant solution and its NMR peak was used as a reference normalized peak for the calculations. It should be noted that some active TADs decomposed over time, without reacting with furan or 2-naphthol, which resulted in a smaller value than the actual value. Theoretically, a maximum of 1.65 mmol of TAD may be present if equimolar ethyl carbazate and triisocyanate are used at the beginning of the synthesis.

**Figure 4:**
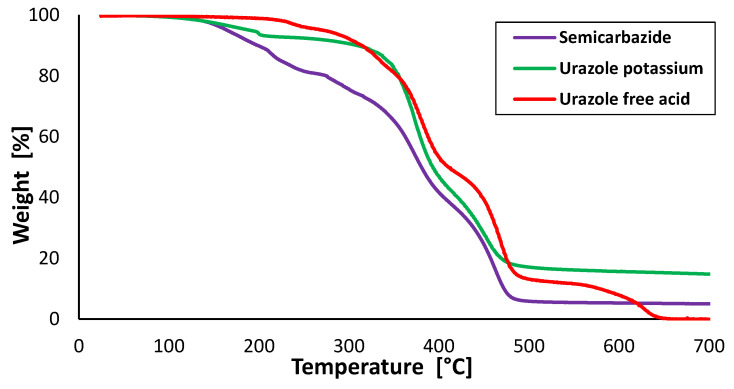
Thermogravimetric analysis of semicarbazide gel, urazole-potassium hydrogel and urazole-free acid gel.

The thermal stability of the hydrogel was analyzed by using thermogravimetric analysis (TGA) which showed that the semicarbazide gel was thermally the least stable (Figure 4). In the urazole formation step, the high temperature probably assisted unreacted isocyanate (i.e., isocyanate remaining in the gel network) to form extra bonds, resulting in higher thermal stability for the urazole gels than the semicarbazide gels. TGA analysis showed that the urazole gel was slightly more stable than when it was in free acid form compared to its potassium salt form below 300 °C. This might be due to the hydrogen bonding capability of the urazoles when they are in free acid form. Under the same conditions, the urazole-potassium salt had a residual mass of 14.85% of its weight after heating to 700 °C, whereas the semicarbazide had 5.01% and the free acid had only 0.05%. The mass contribution of potassium is theoretically 12.90% which explains the high residual mass for the urazole-potassium gel.

**Figure 5:**
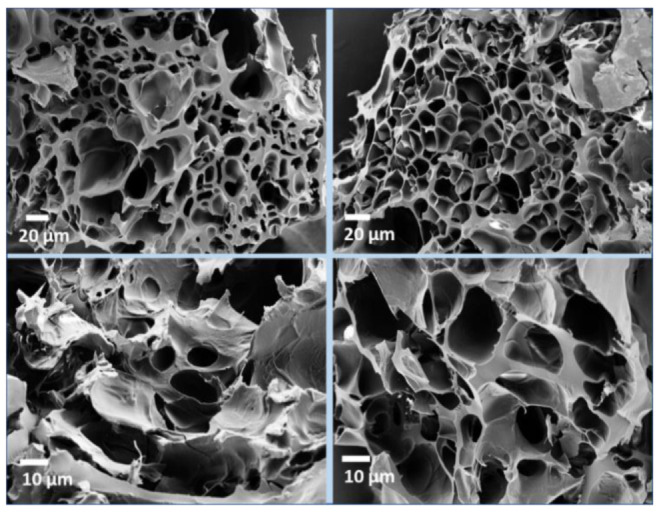
SEM images of freeze dried urazole-potassium hydrogels.

The swelling ratio of the hydrogel was determined as 87% indicating a sponge like character for the material. Scanning electron microscopy (SEM) images showed that the hydrogel indeed had a porous structure like a sponge, with pore sizes between 12 and 30 µm (Figure 5). Images taken from different sections of the gels showed that porosity is homogenous all over the gel. The porosity and pore sizes of the gels can be tuned by changing the curing conditions, such as temperature, initiator amount, or stoichiometric ratio of isocyanate and ethyl carbazate. Generally, a porous structure is a desired property of hydrogels, as it increases the surface area and aids the diffusion of molecules, such as drugs and biologics.

**Figure 6:**
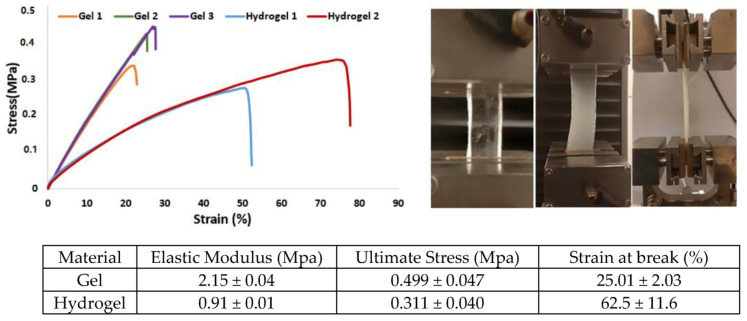
Mechanical analysis results of urazole-potassium hydrogels and semicarbazide gels.

Figure 6 shows the tensile test results for the semicarbazide gel and urazole-potassium hydrogel. The test was conducted when the hydrogels were in a swollen state. Mechanical analysis showed that semicarbazide has a higher elastic modulus than the hydrogel, and it is also more brittle than the hydrogel. However, the hydrogel shows hyperplastic behavior, which is common for hydrogels. The results showed that both gels and hydrogels are consistent with each other, which is a sign of good reproducibility and consistency of material synthesis. The mechanical properties of the samples, such as Young’s modulus and ultimate stress and strain at break, are given in Figure 6. The Young’s moduli of the gel and hydrogel were 2.15 MPa and 0.91 Mpa, respectively. For some applications such as cartilage replacement, the choice of hydrogels is limited because the modulus of most hydrogels is of the order of 0.1 Mpa. Moreover, the gel and hydrogel ultimate stress values are 0.499 Mpa and 0.311 Mpa, respectively and their strain at break values are 25% and 62.5%, respectively. This indicates that the hydrogel strain at break is almost 2.5 times the strain at break for the gel while its ultimate stress is 40% less than the gel. Thus, although the hydrogel became ductile compared to the gel, there was no real drastic decrease in its strength.

**Figure 7:**
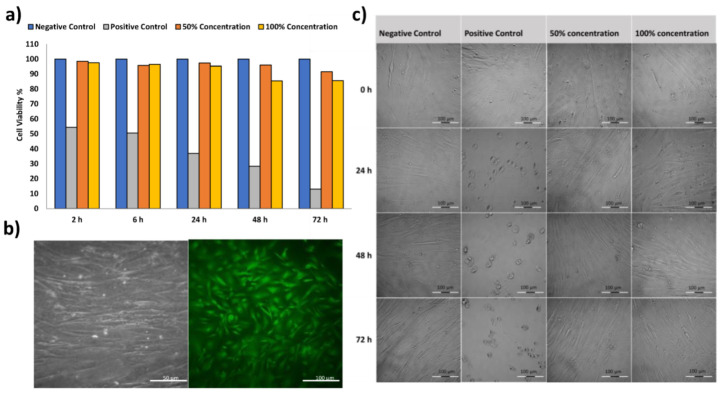
(**a**) Cell viability results at increasing extraction solution concentrations and increasing time. All tests were repeated in four independent incubations for each experiment, and each experiment was repeated three times. (**b**) Light (left) and confocal (right) microscope images of HSF cells on the hydrogel surface at day 3 and (**c**) white light images of cells before the urazole treatment, and on each day after the urazole treatment.

**Figure 8:**
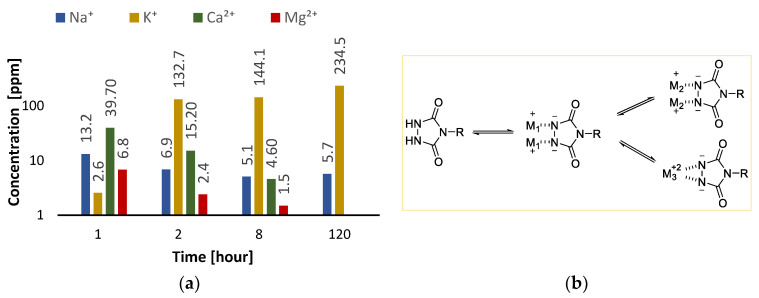
(**a**) Ion-chromatography analysis of tap water treated with urazole-potassium gel for 2, 8, and 120 h. (**b**) Possible ion-exchange mechanism of the urazole group.

Figure 7a shows the results of the WST-1 cell viability assay of human skin fibroblast (HSF) cells which were treated with solutions containing 0.032 mg/mL and 0.016 mg/mL of urazole for 2, 6, 24, 48, and 72 h. As shown in Figure 7a, the hydrogel did not affect the viability of the cells at lower concentrations but reduced cell viability in a dose- and time-dependent manner. Figure 8b shows both white light (left) and confocal microscope images (right) of the cells on the hydrogel containing 0.032 mg/mL of urazole after 72 h of incubation. As can be seen, there was no noticeable change in their morphology. Figure 7c shows a comparison of white light images of the proliferation of the cells treated with the extraction solution at two concentrations for 72 h. Consistent with the viability results, the hydrogel did not exhibit any significant toxicity or affect the cell viability and attachment. Overall, the data in the present study suggest that the prepared hydrogel can be considered to be a biocompatible material in vitro but further in vivo evaluation is needed.

We observed that urazole exchanges potassium ions with other ions in the medium over time, possibly via the mechanism depicted in Figure 8b. It is expected that urazole might selectively bind to higher valent ions owing to its unique structure. Ion-chromatography analysis showed that urazole-potassium gel can remove Ca^2+^ and Mg^2+^ ions completely, and Na^+^ partially from tap water via exchange with K^+^ ions (Figure 8a). This process is important for treating water hardness. We also tested change of water hardness with commercially available water hardness test kits. The results showed that urazole-potassium gel (1% *w*/*w*), reduced water hardness of tap water from 3.6 °dH to 1.3 °dH when the gel was kept in water for 12 h. While after 36 h of keeping the gel inside water, hardness was reduced to 0.1 °dH for the same amount of gel.

## 3. Conclusions

In conclusion, we demonstrated an easy pathway to fabricate a urazole containing hydrogel. It showed superior mechanical properties and good cell viability indicating its biocompatibility, which bodes well for its potential biomedical and ion-exchange applications. Synthesis of urazole-based hydrogels can be made on a large scale from commercially available isocyanates. Finally, novel polyurethane and polyurea based hydrogels can be constructed with the proposed strategy thus opening doors for further investigations.

## 4. Materials and Methods

### 4.1. Materials

All reagents were purchased from Sigma-Aldrich (Saint Louis, MO, USA) unless otherwise noted. Tetrahydrofuran (THF) was purchased as anhydrous and inhibitor free (>99.9%). Poly(hexamethylene diisocyanate)—viscosity 1300–2200 cP (25 °C)—was kept under nitrogen after opening the bottle.

### 4.2. Methods

^1^H-NMR was performed using a Varian 500 MHz spectrometer (Varian, Palo Alto, CA, USA) at ambient temperature. Attenuated total reflection (ATR) FT-IR spectra were recorded using a Perkin-Elmer Spectrum 100 (Buckinghamshire, England) in the region of 4000–650 cm^−1^. Four scans were completed with a resolution of 2 cm^−1^. A background measurement was performed prior to loading the sample onto the ATR for measurement. Raman spectroscopy used a Renishaw inVia Reflex Raman Microscope and Spectrometer. An Nd-YAG laser with a power of 0.5 mW at 532 nm was used for data acquisition and the spectral range was maintained at 300 cm^−1^ to 3000 cm^−1^. A field emission gun SEM (FE-SEM, Zeiss LEO Supra 35VP) was used to observe microstructures of the urazole-potassium hydrogels. The samples were sputter-coated with a thin layer of gold palladium prior to SEM observation. Thermogravimetric analysis (TGA) was performed (Shimadzu, DTG-60H, Kyoto, Japan) from room temperature to 500 °C with a heating rate of 10 °C min^−1^ in a nitrogen atmosphere. Mechanical analysis of the gels and hydrogels was conducted with tensile tests. The tests were performed using an Instron 5982 universal tensile system (Instron, Norwood, MA, USA) with a 200 N static load cell and at a tension rate of 5.0%/min up to failure of the samples under constant temperature and humidity conditions. The material tensile modulus € was determined from the linear portion of the stress–strain curve slope. Moreover, ultimate stress and fracture strain was also reported. Ion chromatography analysis was carried out at the water analysis laboratories of Istanbul Municipality Water and Sewage Administration, Eyup Sultan, Istanbul (ISKI) using Ion Chromatography Spectrometry (Shimadzu, Kyoto, Japan). Na, K, Ca^2+^ were analyzed by EPA 300.1 method. Mg^2+^ was analyzed by SM 3500-Mg B method.

Synthesis of semicarbazide gel: In a typical synthesis, 8.6 mL poly(hexamethylene diisocyanate) was added to an oven dried 100-mL round bottom flask together with 50 mL anhydrous THF under inert conditions. This mixture was stirred for 10 min while keeping the flask in an ice bath. Then, 2.10 g ethyl carbazate was dissolved in 20 mL THF and added to the round bottom flask over 3–5 min in portions. The reaction was kept in stirring mode for 60 more minutes. Next, 100 µL water was added and the solution was poured into glass petri dishes. Then, a watch glass was used to cover it for 2 days to let the THF evaporate slowly. Afterwards, the watch glass was removed when all the THF had evaporated and held for 2 more days. Finally, the gel was peeled from the petri dish (Figure S1). * Note: Video was recorded for slightly different stoichiometric ratios and concentrations of chemicals. Improved synthetic procedure was given above.

Synthesis of urazole gel as potassium salt: In a typical synthesis, 10 g of the semicarbazide gels from the previous step were cut into the pieces and placed into a 100-mL round bottom flask. In method A, 3 g of anhydrous K_2_CO_3_ and anhydrous ethanol was placed and refluxed for 24 h. After that, the gel was placed into beaker and kept in 100 mL water for 20 min. This process was repeated 4 times. Then, the gel was dried under open air for 2–3 days. In method B, gels were placed into aqueous K_2_CO_3_ solution (10% *w*/*w*) and refluxed for 2 days. Finally, the gels were dialyzed against DI water several times to remove K_2_CO_3_ completely.

Synthesis of urazole gel as free acid: The potassium salt version of the urazole gels were placed into DI water. Then, concentrated HCl was added until the pH of the solution became 1. The gels were kept at this pH for 4 h and then dialyzed against DI water several times to remove HCl and KCl completely.

Synthesis of poly(hexamethylene-semicarbazide): In a typical synthesis, 4.3 mL poly(hexamethylene diisocyanate) was added to semicarbazide solution (3.3 g) in 100 mL anhydrous THF under inert conditions. The mixture was stirred for 10 min while keeping the flask in an ice bath. Then, the ice bath was removed, and the reaction was kept under stirring for an hour at ambient temperature. A white polymeric sticky material was precipitated in the reaction flask over time. Then, the THF was decanted off and 100 mL fresh THF was added and stirring continued for 30 min to remove excess semicarbazide. Finally, the THF was decanted, and the material dried under vacuum. Isolated yield: 7.43 g (93%).

Synthesis of poly(hexamethylene-urazole): 2 g of poly(hexamethylene-semicarbazide) was added into an oven dried 100 mL round bottom flask. Then, 1 g of anhydrous K_2_CO_3_ and 60 mL anhydrous ethanol were added. The mixture was refluxed for 24 h. Then, most of the ethanol was decanted carefully and the rest was removed under vacuum. The sticky polymer-K_2_CO_3_ mixture was dissolved in an ice-water mixture. Then, 6M HCl was added slowly, and a white solid was precipitated immediately. More HCl was added until the pH of the solution became 1–2. The product was collected by vacuum filtration and washed with excess DI water to remove acid. The product was dried under vacuum. Isolated yield: 1.61 g.

Synthesis of triazolinedione gel: In a typical synthesis, 200 mg of urazole gel was placed into 3 mL dichloromethane (DCM). Then, 100 µL HNO_3_ was added, and the mixture was shaken vigorously for 3–5 min. Finally, the gels were filtered and washed with excess DCM to remove any residual HNO_3_.

Quantification of urazole content: 150 mg of 2-naphthol was dissolved in 7.5 mL CDCl_3_. To this solution, one drop of acetonitrile was added to be used as an integration reference. One mL aliquots of this solution were added into three batches of the TAD-gel that had been oxidized and washed in the previous 10 min. After 45 min, 0.7 mL of the solutions was taken for NMR analysis. In each spectrum, the acetonitrile peaks were normalized. Loss of 2-naphthol was determined by NMR calculation.

Determination of swelling ratio of hydrogels: Swollen hydrogels were frozen using liquid nitrogen and then freeze dried to complete dryness. Then, the freeze-dried gels were placed into DI water or phosphate buffer (Figure S8). The swelling ratios of the hydrogels were calculated using the formula below.

Swelling ratio = (Mass of the swollen gel − mass of the dried gel)/mass of the dried gel × 100.

Ion-exchange study: 550 mL of tap water was collected into a 1 L Erlenmeyer flask and 50 mL of it was separated and kept as a control. Then 50 g of dry urazole-potassium gel was added to the tap water. After 2, 8, and 120 h, 10 mL aliquots of water were taken for ion-chromatography analysis. Samples were filtered through a 0.22 µm or 0.45 µm filter before analysis.

Cell culture: HSF cells (ATCC, PCS-201-030, Manassas, VA, USA) were incubated at 37 °C under humidified atmospheric air with 5% CO_2_ and cultured in Dulbecco’s modified Eagle’s medium (DMEM, Sigma-Aldrich, Taufkirchen, Germany) supplemented with 5% Penicillin-Streptomycin Ampicillin (PSA), 5% l-glutamine, and 10% fetal bovine serum (FBS) in tissue culture flasks (TPP, Trasadingen, Switzerland). Cells were collected from Trypsin/D-Hanks solution (0.25 *w*/*v*%, Gibco, New York City, NY, USA) until they reach 90% confluency. Collected cells were seeded in a 96-well plate for incubation at a density of 5 × 10^3^ cells/well.

Cell viability assay: Cell viability analysis was performed by using tetrazolium salt WST-1 colorimetric assay after treatment of the hydrogel extract on seeded cells in a 96-well plate. Autoclaved samples were prepared, 0.02 mg based on ISO 10993-12 protocol, and incubated in 1 mL DMEM 72 h at 37 °C at 80 rpm. Seeded cells were cultivated at 100% and 50% extract concentrations., Negative and positive controls were conducted without the material and with 5% DMSO, respectively. After 2 h, 6 h, 24 h, 48 h, and 72 h incubation, each well was rinsed with PBS to clean the complete extraction residue. The treated cells were cultivated with a medium containing %10 WST-1 at 3 h. Consequently, the formazan was converted from the tetrazolium salt in the living cells and absorbance of the medium was measured at 450 nm with an ELISA reader. The cell viability percentage data were calculated with a normalized negative control.

## 5. Patents

A patent application has been submitted with related ion-exchange applications. “Urazole containing materials for ion-exchange applications”. S.B.H., Patent Application Number: TR2021/004015.

## Figures and Tables

**Table 1:** Triazolinedione contents of gels via 2-naphthol loss experiment.

	2-Naphthol Loss ^a^			Active TAD ^b^	
Batch	(%)	mg	mmol	mmol	mg
Gel 1	26.9	5.38	0.037	0.74	76.3
Gel 2	23.6	4.74	0.033	0.66	67.9
Gel 3	25.8	5.18	0.036	0.72	74.2
Average	25.4	5.10	0.035	0.71	72.7

^a^ Determined by ^1^H-NMR for 50 mg gel, ^b^ calculated for 1 g of material.

## Data Availability

Electronic supporting information is available.

## Supplementary Materials

The following are available online at https://www.mdpi.com/article/10.3390/gels7040261/s1, Scheme S1: Synthesis of poly(hexamethylene urazole) and its oxidation to TAD. Oligomers were not shown, Figure S1: (a) Semicarbazide bearing polyurea gel (b) Urazole bearing gel (dried and cut into smaller pieces) (c) Urazole bearing gel swollen in DI water; Figure S2: Bubble free semicarbazide gel. Figure S3: ^1^H-NMR of aniline–PTAD (1:1.1) adduct in CDCl_3_. Peak at 4.55 is for aniline’s NH_2_ protons (after the TAD adduct); Figure S4: Mass spectrum of Aniline–PTAD adduct; Figure S5: ^1^H-NMR spectrum of the PTAD/2-naphthol adduct in dmso-d6, Figure S6: FT-IR spectrum of poly(hm-urazole) free acid and poly(hm-semicarbazide), Figure S7: Raman spectrum of urazole free acid gel, semicarbazide gel and urazole potassium salt gel, Figure S8: Swelling ratio of urazole potassium gel in PBS buffer over time.

## References

[1] Cascone S., Lamberti G. (2020). Hydrogel-based commercial products for biomedical applications: A review. Int. J. Pharm..

[2] Wichterle O., Lim D. (1960). Hydrophilic gels for biological use. Nature.

[3] Kopecek J. (2009). Hydrogels: From soft contact lenses and implants to self-assembled nanomaterials. J. Polym. Sci. Pol. Chem..

[4] Calo E., Khutoryanskiy V.V. (2015). Biomedical applications of hydrogels: A review of patents and commercial products. Eur. Polym. J..

[5] Chen J., Park H., Park K. (1999). Synthesis of superporous hydrogels: Hydrogels with fast swelling and superabsorbent properties. J. Biomed. Mater. Res..

[6] Kamath K.R., Park K. (1993). Biodegradable hydrogels in drug delivery. Adv. Drug Deliv. Rev..

[7] Nicodemus G.D., Bryant S.J. (2008). Cell Encapsulation in Biodegradable Hydrogels for Tissue Engineering Applications. Tissue Eng. Part B Rev..

[8] Baran P.S., Guerrero C.A., Corey E.J. (2003). The first method for protection—Deprotection of the indole 2, 3-π bond. Org. Lett..

[9] Ban H., Gavrilyuk J., Barbas C.F. (2010). Tyrosine bioconjugation through aqueous ene-type reactions: A click-like reaction for tyrosine. J. Am. Chem. Soc..

[10] Billiet S., Bruycker K.D., Driessen F., Goossens H., Speybroeck V.V., Winne J.M., Du Prez F.E. (2014). Triazolinediones enable ultrafast and reversible click chemistry for the design of dynamic polymer systems. Nat. Chem..

[11] Naik A., Alzeer J., Triemer T., Bujalska A., Luedtke N.W. (2017). Chemoselective modification of vinyl DNA by triazolinediones. Angew. Chem..

[12] Hanay S.B., O’Dwyer J., Kimmins S.D., de Oliveira F.C.S., Haugh M.G., O’Brien F.J., Cryan S.A., Heise A. (2018). Facile Approach to Covalent Copolypeptide Hydrogels and Hybrid Organohydrogels. ACS Macro Lett..

[13] Houck H.A., Blasco E., Du Prez F.E., Barner-Kowollik C. (2019). Light-Stabilized Dynamic Materials. J. Am. Chem. Soc..

[14] Mondal P., Jana G., Behera P.K., Chattaraj P.K., Singha N.K. (2020). Fast “ES-Click” Reaction Involving Furfuryl and Triazolinedione Functionalities toward Designing a Healable Polymethacrylate. Macromolecules.

[15] Choghamarani A.G., Chenani Z., Mallakpour S. (2009). Supported Nitric Acid on Silica Gel and Polyvinyl Pyrrolidone (PVP) as an Efficient Oxidizing Agent for the Oxidation of Urazoles and Bis-urazoles. Synth. Commun..

[16] Zolfigol M.A., Zebarjaidan M.H., Chehardoli G., Mallakpour S.E., Shamsipur M. (2001). An efficient method for the oxidation of urazoles with [NO^+^ crown H(NO_3_)_2_^−^]. Tetrahedron.

[17] Rahimi A., Habibi D., Rostami A., Zolfigol M.A., Mallakpour S.E. (2018). Laccase-catalyzed, aerobic oxidative coupling of 4-substituted urazoles with sodium arylsulfinates: Green and mild procedure for the synthesis of arylsulfonyl triazolidinediones. Tetrahedron Lett..

[18] Sato S., Hatano K., Tsushima M., Nakamura H. (2018). 1-Methyl-4-aryl-urazole (MAUra) labels tyrosine in proximity to ruthenium photocatalysts. Chem. Commun..

[19] Keana J.F.W., Guzikowski A.P., Ward D.D., Morat C., Van Nice F.L. (1983). Potent hydrophilic dienophiles synthesis and aqueous stability of several 4 aryl 1 2 4 triazoline 3 5 diones and sulfonated 4 aryl 1 2 4 triazoline 3 5 diones and their immobilization on silica gel. J. Org. Chem..

[20] Laure W., De Bruycker K., Espeel P., Fournier D., Woisel P., Du Prez F.E., Lyskawa J. (2018). Ultrafast Tailoring of Carbon Surfaces via Electrochemically Attached Triazolinediones. Langmuir.

[21] Gordon P.G., Audrieth L.F. (1955). Hydrazine Derivatives of the Carbonic and Thiocarbonic Acids VI. New Synthesis of Urazole. J. Org. Chem..

[22] Ohashi S., Leong K., Matyjaszewski K., Butler G.B. (1980). Ene reaction of triazolinediones with alkenes. 1. Structure and properties of products. J. Org. Chem..

[23] Bausch M.J., David B., Dobrowolski P., Guadalupe-Fasano C., Gostowski R., Selmarten D., Prasad V., Vaughn A., Wang L.H. (1991). Proton-transfer chemistry of urazoles and related imides, amides, and diacyl hydrazides. J. Org. Chem..

[24] Mallakpour S.E., Karami-Dezcho B., Sheikholeslami B. (1998). Polymerization of 1-methyl-2,6-bis[1-(4-phenylurazolyl)]pyrrole dianion with alkyldihalides. Polym. Int..

[25] Sowerby R.L. (1986). Urazole Compositions Useful as Additives for Functional Fluids.

[26] Adams D.R., Barnes A.F., Cassidy F., Thompson M. (1984). The synthesis of 8,10,12-triazaprostaglandin analogues: 1,2,4-triazolidine-3,5-dione derivatives. J. Chem. Soc. Perkin Trans..

[27] Kolb V.M., Dworkin J.P., Miller S.L. (1994). Alternative bases in the RNA world: The prebiotic synthesis of urazole and its ribosides. J. Mol. Evol..

[28] Yang R., Mu W., Chen Q. (2019). Urazole-Au nanocluster as a novel fluorescence probe for curcumin determination and mitochondria imaging. Food Anal. Methods.

[29] Hanay S.B., Ritzen B., Brougham D., Dias A.A., Heise A. (2017). Exploring Tyrosine-Triazolinedione (TAD) Reactions for the Selective Conjugation and Cross-Linking of N-Carboxyanhydride (NCA) Derived Synthetic Copolypeptides. Macromol. Biosci..

[30] Hanay S.B. (2020). Triazolinedione Bearing Gels. ChemRxiv.

