# Supplementary material for: Exploiting Urazole’s Acidity for Fabrication of Hydrogels and Ion-Exchange Materials
Source: Gels. 2021 Dec 13;7(4):261. doi: 10.3390/gels7040261 (PMC8701905; doi:10.3390/gels7040261)
Supplement: Supplementary file 1 [file gels-07-00261-s001.zip › gels-1453668-for conversion-SI.pdf]

gels

Article

Exploiting Urazole's Acidity for Fabrication of Hydrogels and Ion-Exchange Materials

Saltuk B. Hanay [1,2,3,*], Ali Fallah [1], Efsun Senturk [1,3], Zeliha Yetim [4], Ferdows Afghah [1], Hulya Yilmaz [2], Mustafa Culha [2,5], Bahattin Koc [1], Ali Zarrabi [6] and Rajender S. Varma [7,*]

Supporting Information

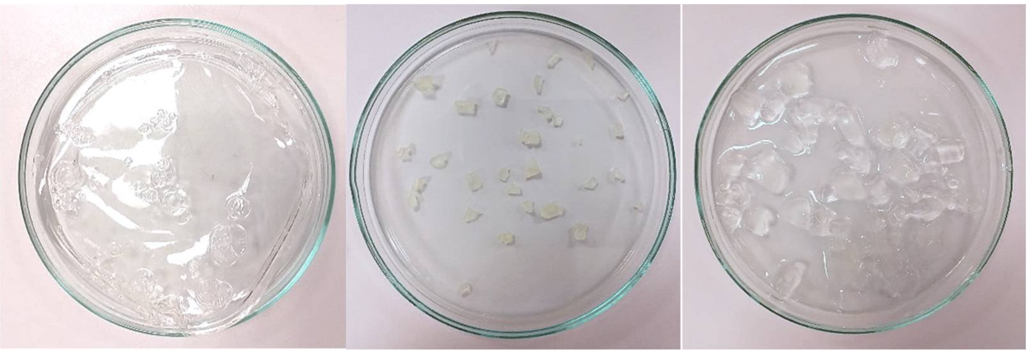

Figure S1. a) Semicarbazide bearing polyurea gel b) Urazole bearing gel (dried and cut into smaller pieces) c) Urazole bearing gel swollen in DI water.

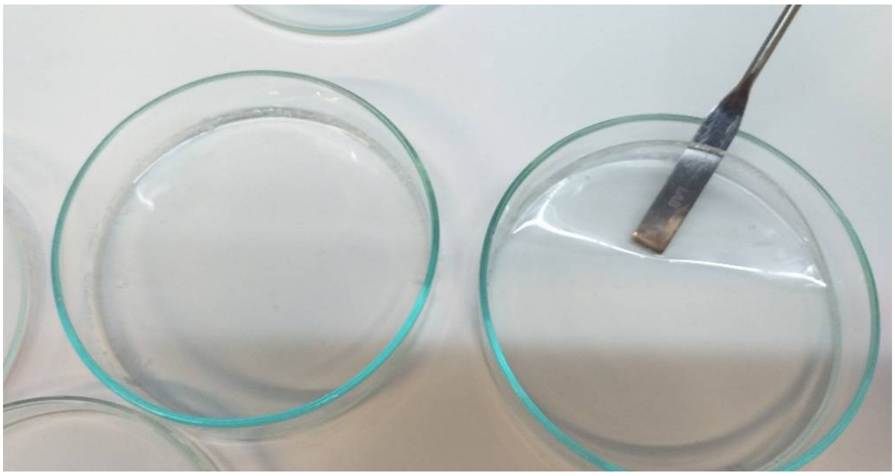

Figure S2. Bubble free semicarbazide gel.

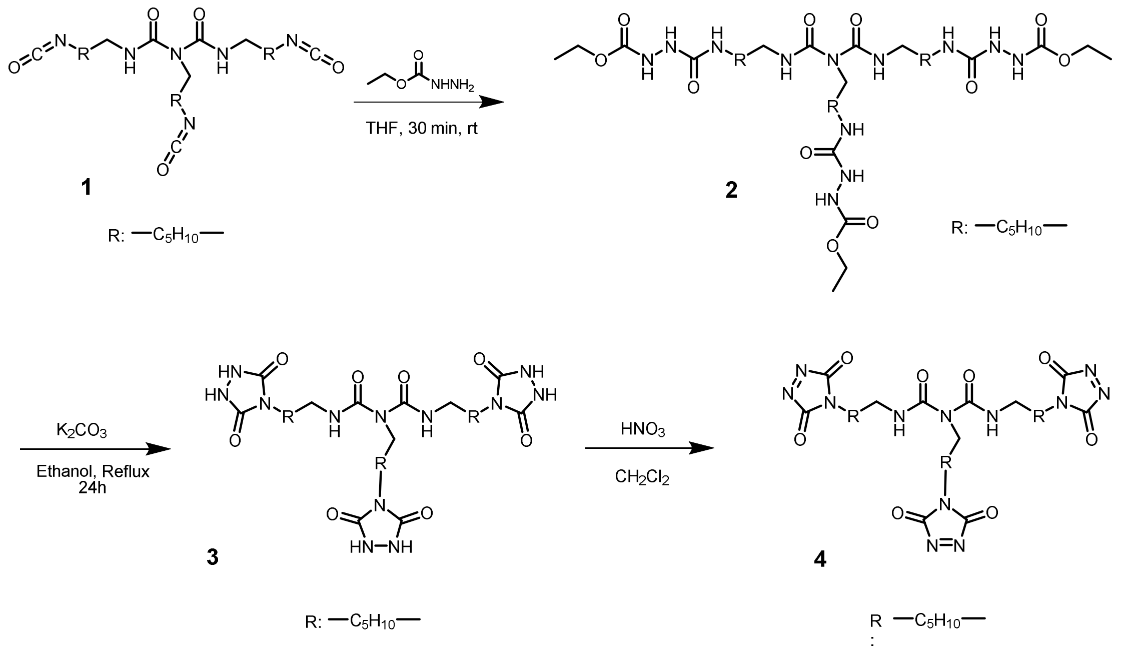

Scheme S1. Synthesis of poly(hexamethylene urazole) and its oxidation to TAD. Oligomers were not shown.

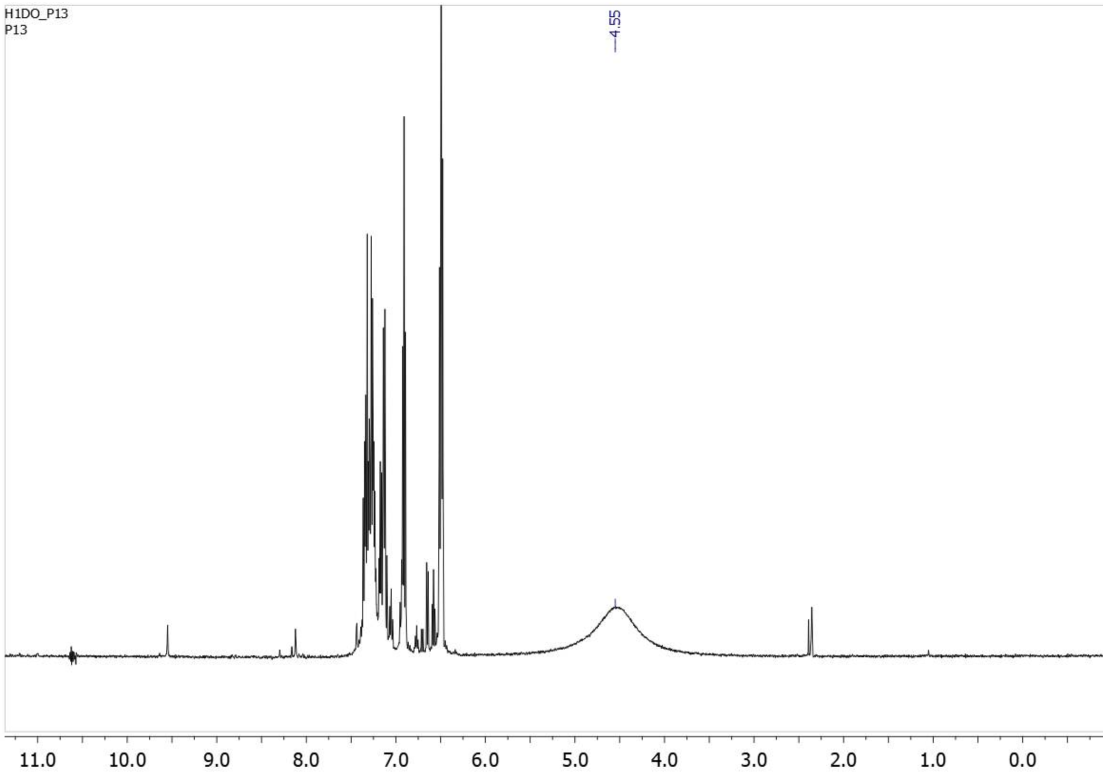

Figure S3. ^{1}H-NMR of aniline – PTAD (1:1.1) adduct in $CDCl_3$. Peak at 4.55 is for aniline's NH_2 protons (after the TAD adduct)

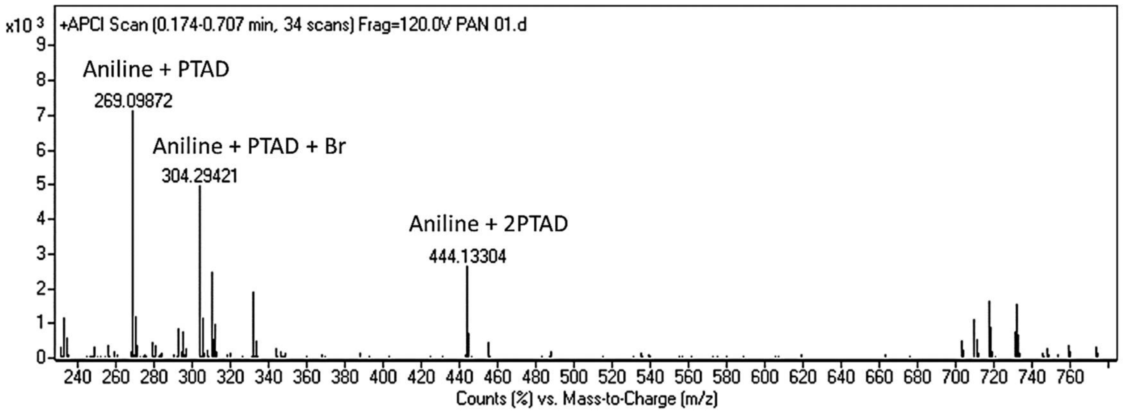

Figure S4. Mass spectrum of Aniline – PTAD adduct.

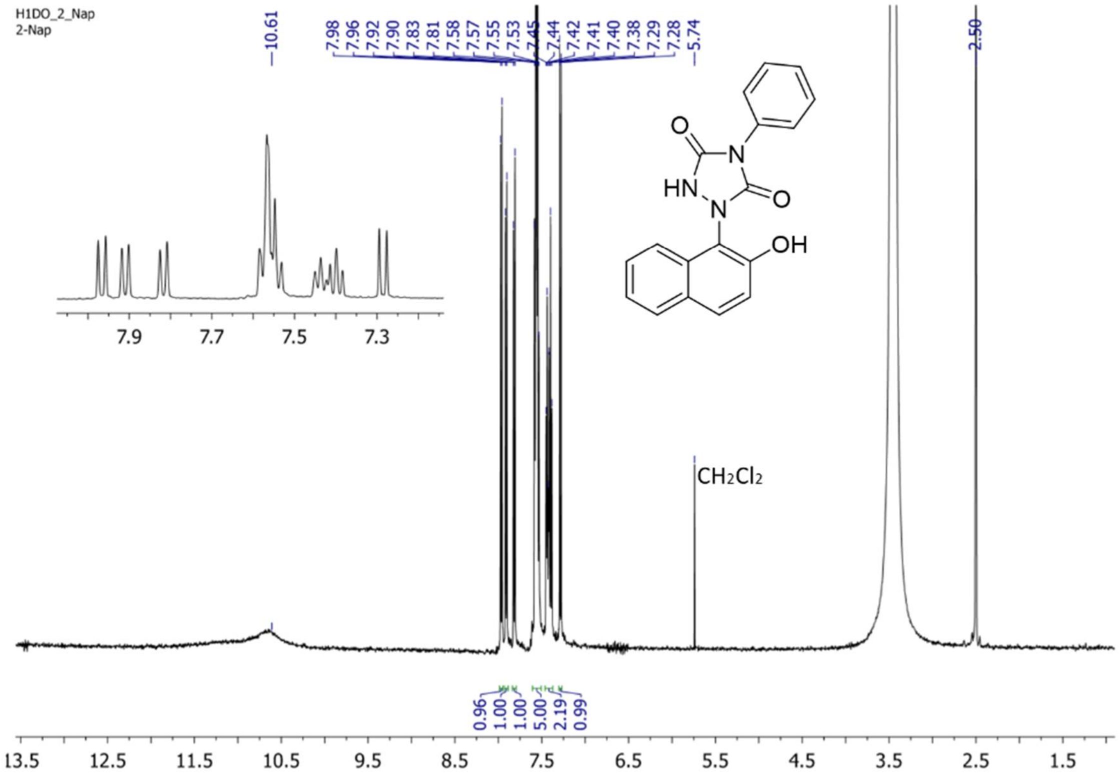

Figure S5. ^{1}H-NMR spectrum of the PTAD/2-naphthol adduct in dmso-d6.

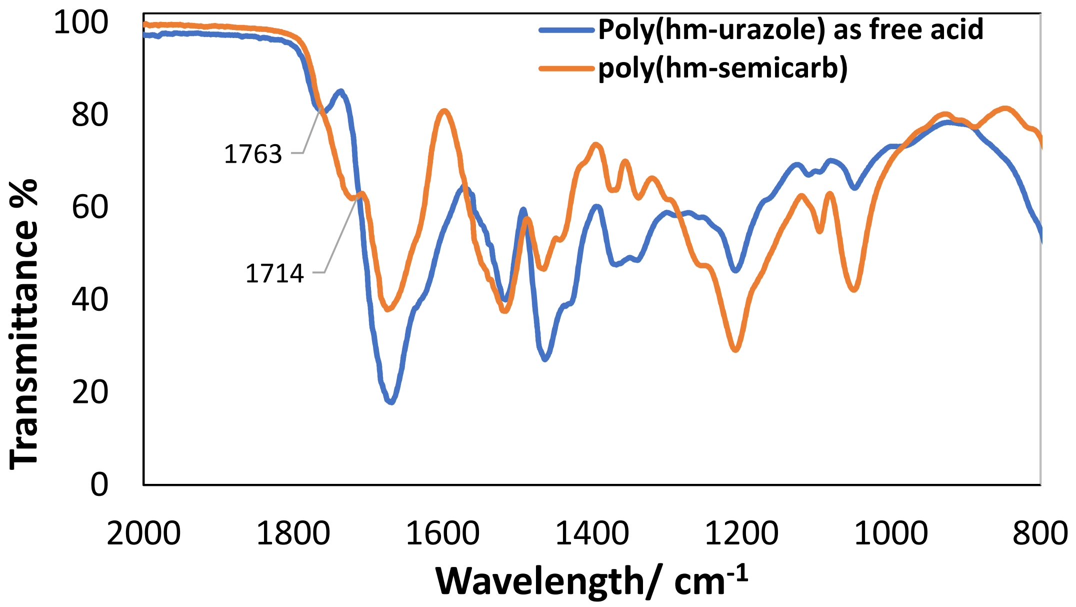

Figure S6. FT-IR spectrum of poly(hm-urazole) free acid and poly(hm-semicarbazide).

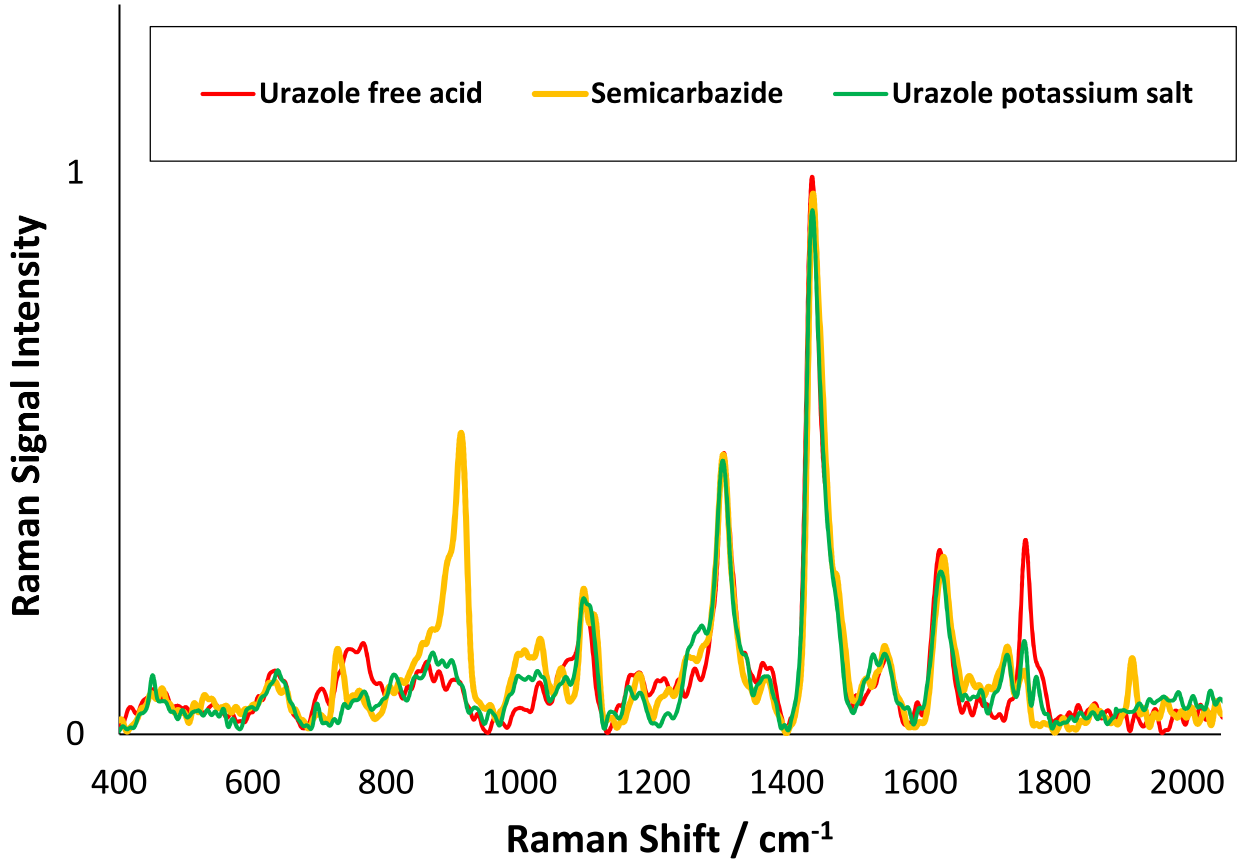

Figure S7. Raman spectrum of urazole free acid gel, semicarbazide gel and urazole potassium salt gel.

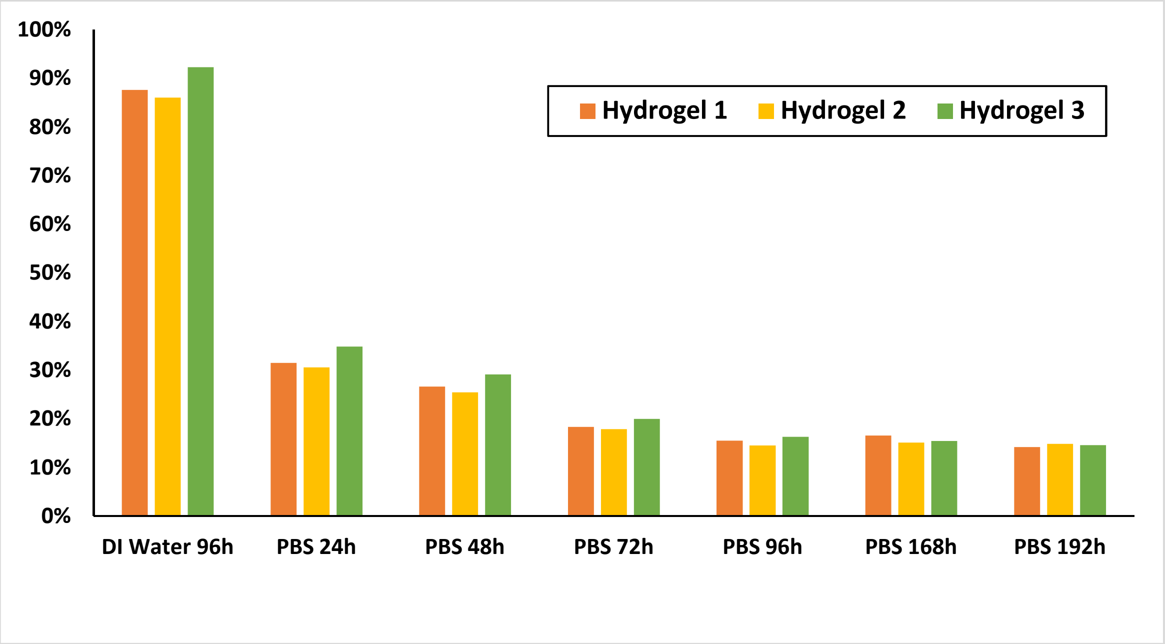

Figure S8. Swelling ratio of urazole potassium gel in PBS buffer over time.
